# Factors determining dengue outbreak in Malaysia

**DOI:** 10.1371/journal.pone.0193326

**Published:** 2018-02-23

**Authors:** Rohani Ahmad, Ismail Suzilah, Wan Mohamad Ali Wan Najdah, Omar Topek, Ibrahim Mustafakamal, Han Lim Lee

**Affiliations:** 1 Medical Entomology Unit & WHO Collaborating Centre for Vectors, Institute for Medical Research, Kuala Lumpur, Malaysia; 2 School of Quantitative Sciences, Universiti Utara Malaysia, Sintok, Kedah, Malaysia; 3 Parasitology Department, Faculty of Medicine, University of Malaya, Kuala Lumpur, Malaysia; 4 Disease Control Division, Ministry of Health, Putrajaya, Malaysia; 5 Selangor State Health Department, Shah Alam, Selangor, Malaysia; National Taiwan Ocean University, TAIWAN

## Abstract

A large scale study was conducted to elucidate the true relationship among entomological, epidemiological and environmental factors that contributed to dengue outbreak in Malaysia. Two large areas (Selayang and Bandar Baru Bangi) were selected in this study based on five consecutive years of high dengue cases. Entomological data were collected using ovitraps where the number of larvae was used to reflect *Aedes* mosquito population size; followed by RT-PCR screening to detect and serotype dengue virus in mosquitoes. Notified cases, date of disease onset, and number and type of the interventions were used as epidemiological endpoint, while rainfall, temperature, relative humidity and air pollution index (API) were indicators for environmental data. The field study was conducted during 81 weeks of data collection. Correlation and Autoregressive Distributed Lag Model were used to determine the relationship. The study showed that, notified cases were indirectly related with the environmental data, but shifted one week, i.e. last 3 weeks positive PCR; last 4 weeks rainfall; last 3 weeks maximum relative humidity; last 3 weeks minimum and maximum temperature; and last 4 weeks air pollution index (API), respectively. Notified cases were also related with next week intervention, while conventional intervention only happened 4 weeks after larvae were found, indicating ample time for dengue transmission. Based on a significant relationship among the three factors (epidemiological, entomological and environmental), estimated Autoregressive Distributed Lag (ADL) model for both locations produced high accuracy 84.9% for Selayang and 84.1% for Bandar Baru Bangi in predicting the actual notified cases. Hence, such model can be used in forestalling dengue outbreak and acts as an early warning system. The existence of relationships among the entomological, epidemiological and environmental factors can be used to build an early warning system for the prediction of dengue outbreak so that preventive interventions can be taken early to avert the outbreaks.

## Introduction

Dengue incidence has increased dramatically around the world in recent decades. About half of the world’s population is now at risk, with the number of reported cases increasing from 2.2 million in 2010 to 3.2 million in 2015 [1]. Dengue is also considered as the most rapidly spreading mosquito-borne viral disease in the world. The disease is now endemic in more than 100 countries, which include South-East Asia as one of the most seriously affected regions. It is one of the most significant vector-borne diseases of humans in terms of global morbidity and mortality [2]; with its more complicated and fatal disease form, the severe dengue, transmitted by the dengue mosquitoes, *Aedes aegypti* (Linnaeus, 1762) and *Ae*. *albopictus* (Skuse, 1895). In Malaysia, a total of 101,357 dengue cases and 237 deaths were reported in 2016 [3]. Most of the cases came from Selangor with 48,491 dengue cases or 51.3% of the total recorded dengue cases in Malaysia [3].

*Aedes aegypti* is considered as the principal species involved in the transmission of dengue viruses in humans [3,4]. Its ability as an efficient vector of dengue virus is explained by its ability to adapt to the different environments; its distinct preference for human habitats and skip-oviposition behaviors [5,6]. Female *Ae*. *aegypti* mainly feed on human host blood, thus results in frequent contacts between the vector and human. This anthropophilic tendency is postulated to be a factor that renders *Ae*. *aegypti* to be more competent in spreading the dengue virus than *Ae*. *albopictus* that feeds on both human and animal blood [3,5]. A female *Ae*. *aegypti* takes multiple blood meals [7] during each egg-laying cycle, increasing the opportunities to acquire and transmit the dengue virus. This species also feeds during daytime, when humans are active. This often leads to interrupted feedings that could further contribute to the number of human hosts that get in contact with the mosquito.

*Aedes albopictus* is a secondary vector of dengue virus in Southeast Asia but has also been documented as the sole vector during some outbreaks where *Ae*. *aegypti* was not present [8]. It is believed that this species is responsible for maintaining the dengue virus in the environment. It is primarily a forest species that has become adapted to rural, suburban and urban human areas in Malaysia, which overlap with the distribution of *Ae*. *aegypti* [9]. This species prefers the outdoor environment for activity and rest, but have also been noted to bite and rest indoors [4,10].

A dengue outbreak prediction study was conducted by the Institute for Medical Research from 2007 until 2009, with the aim of identifying factors contributing to dengue outbreak in Malaysia by focusing on three major aspects—entomological, epidemiological and environmental [11]. A field study was implemented at four dengue prone areas in Kuala Lumpur, Pahang, Kedah and Johor to collect and analyse various parameters to model dengue transmission and outbreak. Ovitraps were located outdoor and monitored weekly for 87 weeks in representing vector population in each area. The effects of environmental parameters on vector breeding were estimated using weather stations (i.e. containing temperature and relative humidity data logger and automated rain gauge) situated at the centre locations in each study site. The relationships between the factors were measured using correlation and Autoregressive Distributed Lag (ADL) model [12]. The findings revealed that last week rainfall, maximum relative humidity and temperature significantly contributed to the increment of the mosquito population. But, the secondary data of rainfall, temperature and relative humidity obtained from the meteorological department disclosed no relationship with mosquito population. This was due to very localized rainfall behaviour commonly occurring in Malaysia. The study also found a good model for each of the studied localities which can be used as a prediction for dengue outbreak in Malaysia [11]. However, the significant relationship only existed between entomological (number of larvae) and environmental factors (rainfall, temperature and relative humidity). There was no significant relationship between epidemiological (notified and onset date) with entomological (number of larvae) and environmental factors (rainfall, temperature and relative humidity) respectively. This was due to the small size of the localities involved in that study, and hence with only a low number of dengue cases which led to difficulty in analyzing the relationship.

Therefore, a large scale study was conducted to determine the contribution of three major factors, namely entomological, epidemiological and environmental factors related to dengue outbreak in Malaysia. The purpose of this study was to replicate previous studies [11] but at two different larger locations in Selangor by additional PCR screening and air pollution index screening and to correlate those data with entomological and environmental factors respectively.

## Materials and methods

Two study areas were selected based on five consecutive years of high dengue cases in Selangor, namely Selayang (N3.249°, E101.668°) and Bandar Baru Bangi (N2.949°, E101.775°). The study was conducted in public residential areas. The funder (Ministry of Health Malaysia) is one of the responsible authority to conduct a study for human health benefits in this areas. Therefor no permission was required. The field study did not involve endangered or protected species. The study was conducted from April 2014 until November 2015, which comprised 81 weeks of data collection based on the three major factors (entomological, environmental and epidemiological).

Entomological data were collected using ovitraps where number of larvae were used to reflect *Aedes* mosquito population size; followed by RT-PCR screening to detect and serotype dengue virus in mosquito. Fifty (50) and 55 ovitraps were placed in Selayang and Bandar Baru Bangi, respectively, whereby the number of ovitraps were determined based on three weeks of pilot survey. Ovitraps have been used as a standard tool in studies on mosquitoes [13,14]. An ovitrap consists of a plastic container of 7 cm diameter and 9 cm in height, with black walls. An oviposition paddle made from hardboard (10 cm × 3.0 cm × 2.5 cm) was placed into each ovitrap with the rough surface upwards. Each ovitrap was filled with tap water to a level of 5.5 cm. After 7 days, all ovitraps were collected and replaced with fresh ovitrap and paddle. Ovitraps were set weekly for 81 weeks and lost or damaged ones were recorded and replaced.

Ovitraps were brought to the laboratory and the contents were poured into a plastic container filled with seasoned water and the eggs/larvae were allowed to further develop in the laboratory. Primary (1°) identification was conducted during which 4th instar larvae were picked up and identified using standard IMR taxonomy keys under a compound microscope. Identified mosquito larvae were segregated according to species, site and date. Paddles were air dried and soaked in the same ovitrap by adding seasoned water after 24 hours. The following 5 days, secondary (2°) identification was done. Water and paddle in each ovitrap were poured again into the same plastic container. Tertiary (3°) identification was conducted after another five days. Larvae of *Ae*. *aegypti* and *Ae*. *albopictus* were pooled with maximum of 20 larvae per pool and stored in freezer at -70°C for dengue virus detection using Reverse Transcriptase-Polymerase Chain Reaction (RT-PCR).

A total of twenty (20) mosquito larvae were pooled in a nuclease-free 1.5 ml micro centrifuge tube. 210 μl of nuclease-free, double-distilled water was added and the mosquito larvae were homogenized on ice using a homogenizer attached to a Pellet Pestle Motor (Kontes, Japan). The homogenized samples were then centrifuged at 5000 x g for 10 minutes at 4°C. QIAmp Viral RNA Mini Kit (Qiagen) was used to extract the viral RNA from the mosquito larva homogenates following the manufacturer’s guidelines. Extracted RNA was kept at -80°C until used.

The RT-PCR method of Lanciotti *et al*. [15] was employed using the dengue universal primers of TCAATATGCTGAAACGCGCGAGAAACCG and TTGCACCAACAGTCAATGTCTTCAGCTTC. Each reaction contained 10.25 μl of nuclease-free water, 2 μl of dNTP mixture, 1.25 μl of dithiotreitol, 0.5 μl of RNAse inhibitor, 0.5 μl of each dengue primer and 0.5 μl of RNA. The reaction was carried out at 51°C for 30 minutes to create cDNA, which was then amplified by the following PCR steps: initial denaturation at 92°C for three minutes, 41 cycles of 92°C for 30 seconds, 51°C for 45 seconds and 72°C for one minute; followed by 72°C for five minutes. For every RT-PCR, a positive control and negative control was included. PCR products were analysed by performing electrophoresis in 2.0% Nusieve PCR gel (FC Bio, USA) at 100 volts and staining with ethidium bromide. The gel was viewed under ultraviolet illuminator (Ultra Lum Inc, California, USA) and the resulting bands were photographed with a Polaroid camera.

**Fig 1:**
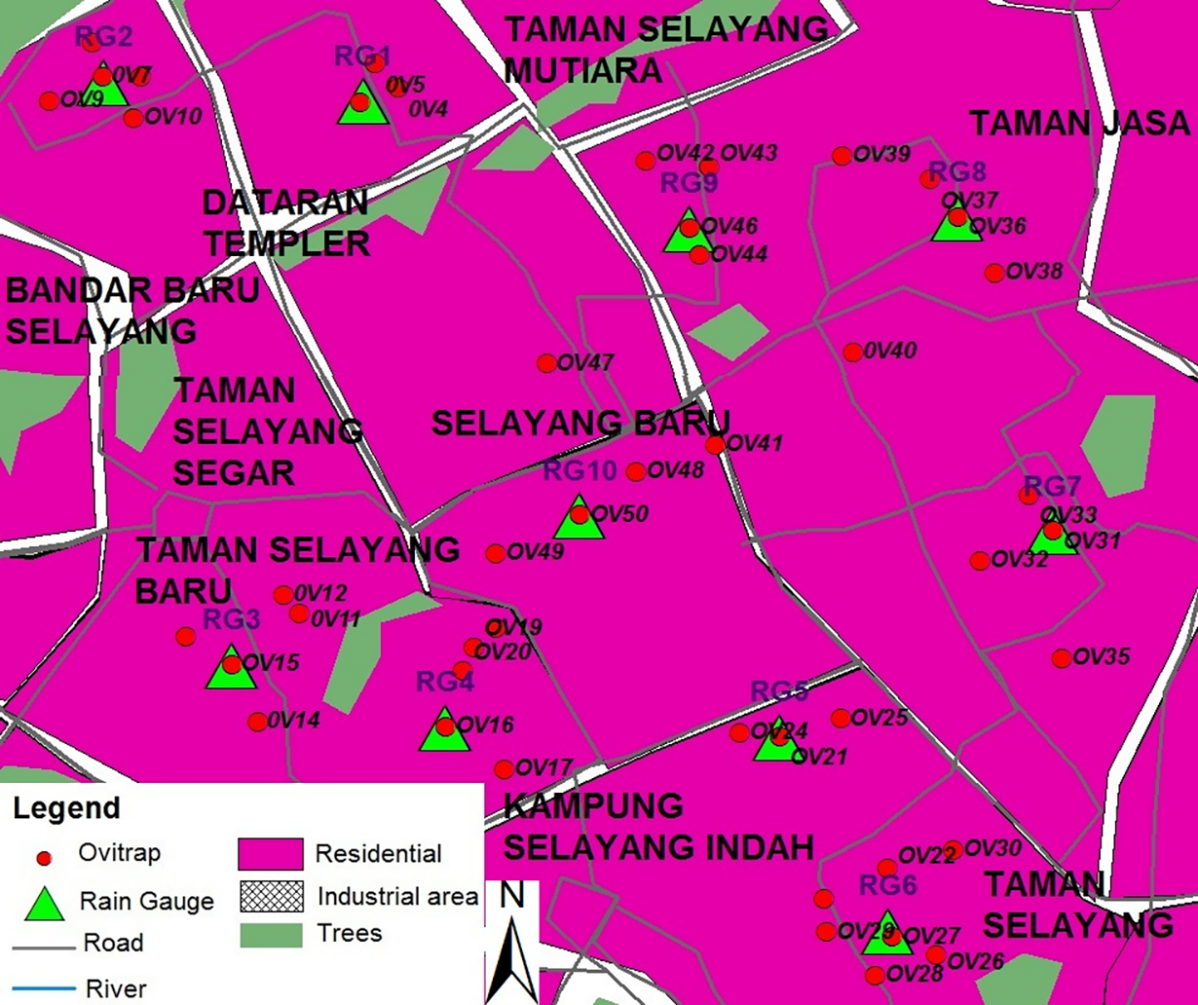
Location of rain gauge and ovitrap (Selayang).

**Fig 2:**
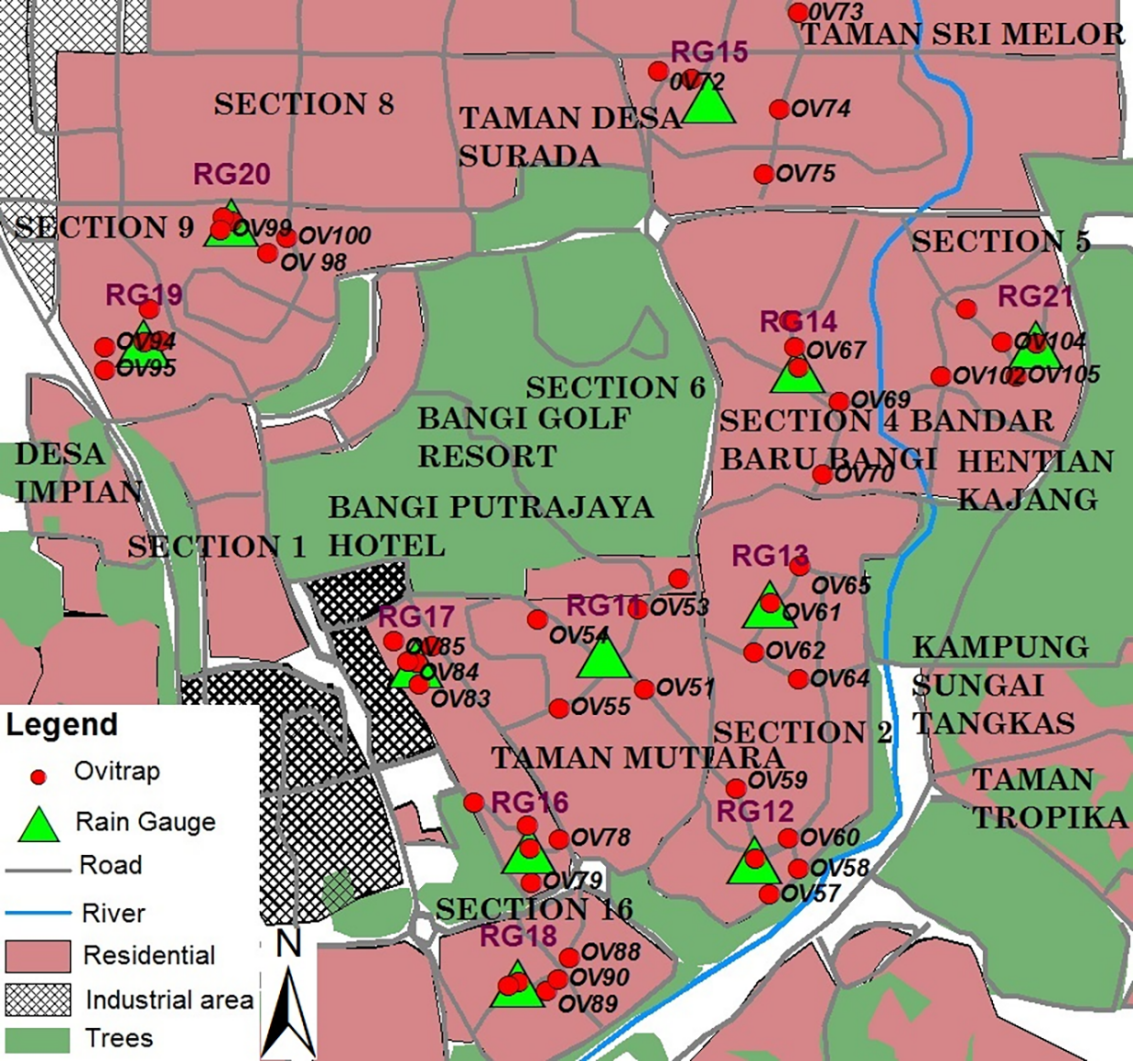
Location of rain gauge and ovitrap (Bandar Baru Bangi).

Rainfall, temperature, relative humidity and air pollution index are indicators for environmental data. Based on a previous study [11], the rainfall appears to be localized in Malaysia. Thus, in this study since it involved large areas, 10 and 11 rain gauges were installed at Selayang and Bandar Baru Bangi, respectively, as indicated in Figs 1 and 2. Five ovitraps were located around each rain gauge together with one set of temperature and relative humidity data logger. In this study air pollution index is included due to frequently occurring haze in Selangor and the API data were obtained from the Department of Environment Malaysia.

Epidemiological data of notified cases, disease onset date, and number of intervention were retrieved from e-Dengue system. It is a web based GIS system used by the Ministry of Health Malaysia to manage data collection on epidemiology, prevention and control, and health promotion activities. A notified case is a case compatible with the clinical description and reported to the nearest health office. The date of notification is the date of the case being reported to the health office, while the date of onset is the date of the first day of a case having fever.

Spearman correlation was used due to the violation of Pearson correlation assumption and followed with Autoregressive Distributed Lag (ADL) model [as defined in Eq (1)] in capturing the lags (weeks) effect in the relationship. The analysis was conducted using SPSS version 22 (Automatic Linear Modeling).

$$y_{it}=\alpha_{i0}+\sum_{j=1}^{J}\alpha_{ij}y_{i(t-j)}+\sum_{k=1}^{K}\sum_{j=0}^{J_{k}}\phi_{ikj}x_{ik(t-j)}+\varepsilon_{it}$$ (1)

where *j* is the lag length, *i* = 1,2,…,N, *t* = 1,2,…,T (time periods) and *y*_*it*_ is the target variable which is the notified cases. *x*_*ikt*_ is the predictors which are epidemiological, entomological and environmental variables.*ε*_*it*_ are identically independently distributed random errors with mean zero and variance $\sigma_{\varepsilon_{it}}^{2}$, *α* and *ϕ* are unknown parameters to be estimated using Ordinary Least Squares (OLS). Four lags for each of the variables was also included.

## Results and discussion

We studied the relationship between dengue infection in mosquitoes with their population density, and the main weather variables of temperature, relative humidity and precipitation as well as their influence on dengue cases. Table 1 displays the correlation values between epidemiological and entomological variables. Selayang and Bandar Baru Bangi have strong correlation between notified cases and this week and last week onset date (Onset, Onset_1) respectively, because 51% of the cases were notified within 3 days from the date of onset and another 49% were notified more than 3 days from the date of onset.

**Table 1:** Correlation of epidemiological and entomological variables.

Location	Notified			Larvae	Notified	Notified	Larvae
	Onset	Onset_1	InterventLD1		Larvae_3	PCR_3	PCR
**Selayang**	0.782***	0.814***	0.533***	Intervention_4 0.265**	0.847***	0.537***	0.568***
**Bandar Baru Bangi**	0.819***	0.811***	0.304***	Intervention_1 -0.218**	0.747***	0.407***	0.436***

*** Significant at 1%.

** Significant at 5%.

Notified ~ this week notified cases.

Onset ~ this week onset date.

Onset_1~ last week onset date.

Larvae ~ this week total larvae.

Larvae_3 ~ last 3 weeks total larvae.

PCR ~ this week positive PCR.

PCR_3 ~ last 3 weeks positive PCR.

InterventLD1 ~ next week intervention.

Intervention_1 & _4 ~ this week & last 4 weeks intervention.

Moderate (0.533) and weak (0.304) correlation were obtained between this week notified cases (notified) with next week intervention (InterventLD1), respectively, for Selayang and Bandar Baru Bangi (Table 1), indicating the increase in this week notified cases increased next week intervention. In other words, next week intervention depends on this week notified cases. Thus, an early control intervention is not possible especially in an area with abundance of cases, such as Selayang and Bandar Baru Bangi resulting in a slight delay in carrying out the intervention activities.

There were weak correlations between larvae and last week intervention for Bandar Baru Bangi and larvae with last 4 weeks intervention for Selayang. The correlations were weak because this study involved large areas and intervention only was only implemented at notified case houses within the range of a 200 m buffer zone. Negative correlation (-0.218) in Bandar Baru Bangi indicated last week intervention reduced this week larvae but Selayang had positive relationship (0.265) which showed the increment of last 4 weeks intervention increased this week larvae. This interesting finding was due to the fact that once massive intervention 4 weeks ago was conducted, then the intervention stopped. An increase of this week’s larvae resulted because the intervention was based on notified cases and not on the number of larvae.

Table 1 also shows that both locations have strong (0.847 and 0.747) correlations between notified cases and last 3 weeks larvae. This corresponds to the expectation that in the presence of an initial viraemic human case, the presence of sufficient mosquito density in a locality allows for dengue transmission if no vector control has been carried out. We then can expect a subsequent initial increase in dengue cases being notified from the locality 3–4 weeks later. This is based on the expected minimum of 14–29 number of days for dengue cases from further transmission to be notified later; after considering that the dengue virus’s extrinsic incubation period is 8–10 days the *Aedes* vectors, the intrinsic incubation period in human is 4–13 days and most (78%) of the dengue cases from the study localities were notified 2–6 days after onset. Thus, when the trend of dengue vector population is increasing, it is crucial that the programme managers especially those in endemic areas to institute prompt vector control actions to prevent an impending surge in dengue cases.

**Fig 3:**
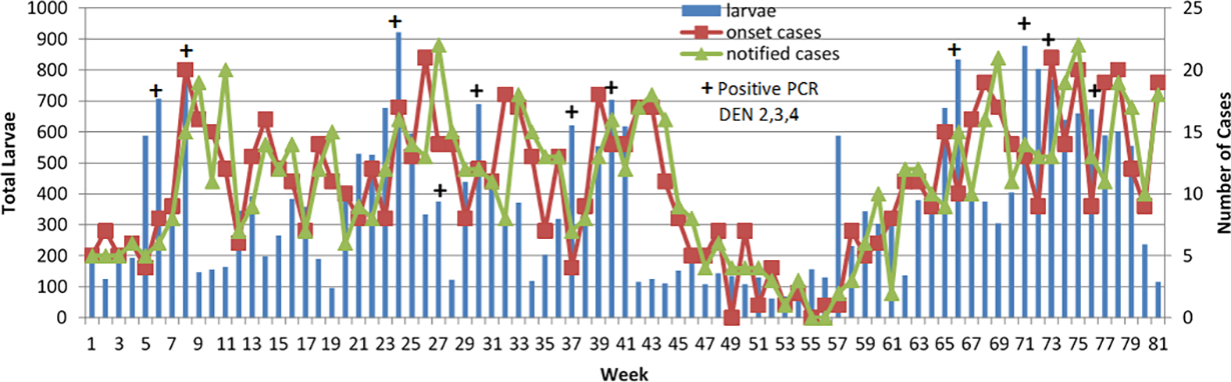
Trend of epidemiological and entomological variables (Selayang).

**Fig 4:**
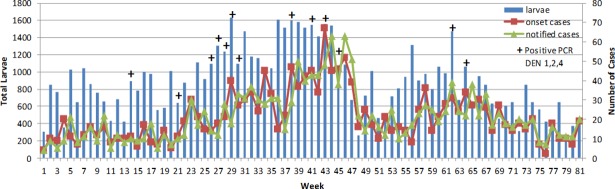
Trend of epidemiological and entomological variables (Bandar Baru Bangi).

There was a moderate correlation (0.537, Selayang and 0.407, Bandar Baru Bangi) between notified and last 3 weeks positive PCR in mosquito (Table 1) because positive PCR was influenced by total larvae collected, as indicated by the correlation value between larvae and PCR (0.568). Figs 3 and 4 showed that when large numbers of larvae were collected, there were increasing chances of obtaining positive PCR. Figs 3 and 4 also revealed the relationship between epidemiological and entomological variables. High number of last 3 weeks positive PCR increased the number of this week notified cases for both locations.

Our study are in general agreement with the implementation of dengue control strategies guided by entomological indices, i.e. a higher number of mosquito vectors increased the risk of dengue occurrence; hence, higher dengue cases should be expected in places with higher density of mosquitoes. Scot & Morrison [16] also showed that, traditional larval indices in Peru correlated with prevalence of human dengue infection. However, in contrast, Pena-Garcia et al [17] did not find a direct relationship between mosquito density and dengue infection in adult mosquito populations they sampled in Colombia.

The existence of many viraemic symptomatic and mildly symptomatic cases among others are the factors that may hamper the prediction of a dengue outbreak; the time interval between the detection of emerging adults to the appearance of clinical cases. Still, Lee & Rohani [18] indicated that, the interval between transovarial dengue virus detection and first human cases ranged from 7 to 41 days, whereas Chow et al, [19] showed that by detecting dengue virus in adult mosquitoes using RT-PCR, it was possible to predict an outbreak six weeks in advance of the occurrence of human cases in Singapore.

The probability of transmission will be low in an area regardless of the magnitude of measures of entomological risk, if human herd immunity is high. Conversely, if herd immunity is low, relative low population densities of *Ae*. *aegypti* could precipitate an epidemic. Moreover, *Ae*. *aegypti* survives and efficiently transmits dengue virus even when their population densities are remarkably low [20]. Various researchers have investigated the relationship between dengue transmission and the *Aedes* population, expressed as larval [21–23] pupal [24–26] and adult indices [27].

Since there are strong correlations between notified cases and last 3 weeks larvae, the next step is to identify the relationship between larvae (entomological) with environmental variables as displayed in Table 2. There were strong (0.799) and moderate (0.549) correlation between larvae and last week rainfall (rainfall_1) for Selayang and Bandar Baru Bangi, respectively. There was moderate (0.688 and 0.546) correlation between larvae and maximum relative humidity but no correlation or weak (0.341) correlation with minimum relative humidity. In addition, there were moderate negative correlations with minimum and maximum temperature. These findings are similar to a previous study by Rohani et al [11] as they mentioned that important role in mosquito breeding was provided by last week rainfall, maximum relative humidity and temperature. The recent frequent occurrence of haze in Selangor motivated us to study the relationship between larvae and air pollution index (API). There are moderate negative correlations (-0.691 and -0.411) between larvae and last week API, indicating that last week high API reduced this week larvae, probably because API caused a reduction of that week adult mosquito, hence this week larvae were reduced.

**Table 2:** Correlation between entomological and environmental variables.

Location	Larvae					
	Rainfall_1	MinTemp	MaxTemp	MinHumid	MaxHumid	API_1
**Selayang**	0.799***	-0.435***	-0.471***	-	0.688***	-0.691***
**Bandar** **Baru** **Bangi**	0.549***	-0.404***	-0.518***	0.341***	0.546***	-0.411**

*** Significant at 1%.

** Significant at 5%.

Larvae ~ this week larvae.

Rainfall_1 ~ last week rainfall.

MinTemp ~ this week minimum temperature.

MaxTemp ~ this week maximum temperature.

MinHumid ~ this week minimum humidity.

MaxHumid ~ this week maximum humidity.

API_1 ~ last week air pollution index.

Our findings with respect to effect of haze on mosquitoes are in general agreement with Massad et al. [28]. They concluded that the fewer than expected number of dengue cases in Singapore in 2006 was caused by an increase in mosquito mortality due to the disproportionate haze affecting the country that year. On the contrary, Wilder-Smith et al., [29] reported no effect of the haze on dengue activity, and even if haze did have an effect on increasing the mortality of mosquitoes, in most years the duration of haze was too short to result in a major effect on dengue case numbers. However, relative humidity, temperature and API are highly related with rainfall. No rain for consecutive 2–3 weeks will reduce the larvae. Furthermore, Malaysia has an equatorial climate–being hot and humid, where rain happen on average at 20 days per month and 70% throughout the year [30].

We are also interested to determine the relationship between notified cases and environmental variables because the existence of these relationships can be used in developing dengue outbreak forecasting model. Table 3 displays a significant correlation between notified cases and last 4 weeks rainfall; last 3 weeks minimum, maximum temperature and relative humidity, respectively, and last 4 weeks API. These relationships aligned with the findings based on Table 2 as explained earlier, where this week larvae were correlated with last week rainfall, this week minimum, maximum temperature, relative humidity; and last week API. Since last 3 weeks larvae correlated with this week notified cases, therefore the relationship between notified and environmental data shifted one week.

**Table 3:** Correlation between epidemiological and environmental variables.

Location	Notified					
	Rainfall_4	MinTemp_3	MaxTemp_3	MinHumid_3	MaxHumid_3	API_4
**Selayang**	0.678***	-0.453***	-0.452***	-	0.674***	-0.637***
**Bandar** **Baru** **Bangi**	0.678***	-0.379***	-0.474***	0.386***	0.656***	-0.393**

*** Significant at 1%.

** Significant at 5%.

Notified ~ this week notified cases.

Rainfall_4 ~ last 4 week rainfall.

MinTemp_3 ~ last 3 week minimum temperature.

MaxTemp_3 ~ last 3 week maximum temperature.

MinHumid_3 ~ last 3 week minimum humidity.

MaxHumid_3 ~ last 3 week maximum humidity.

API_4 ~ last 4 week air pollution index.

**Fig 5:**
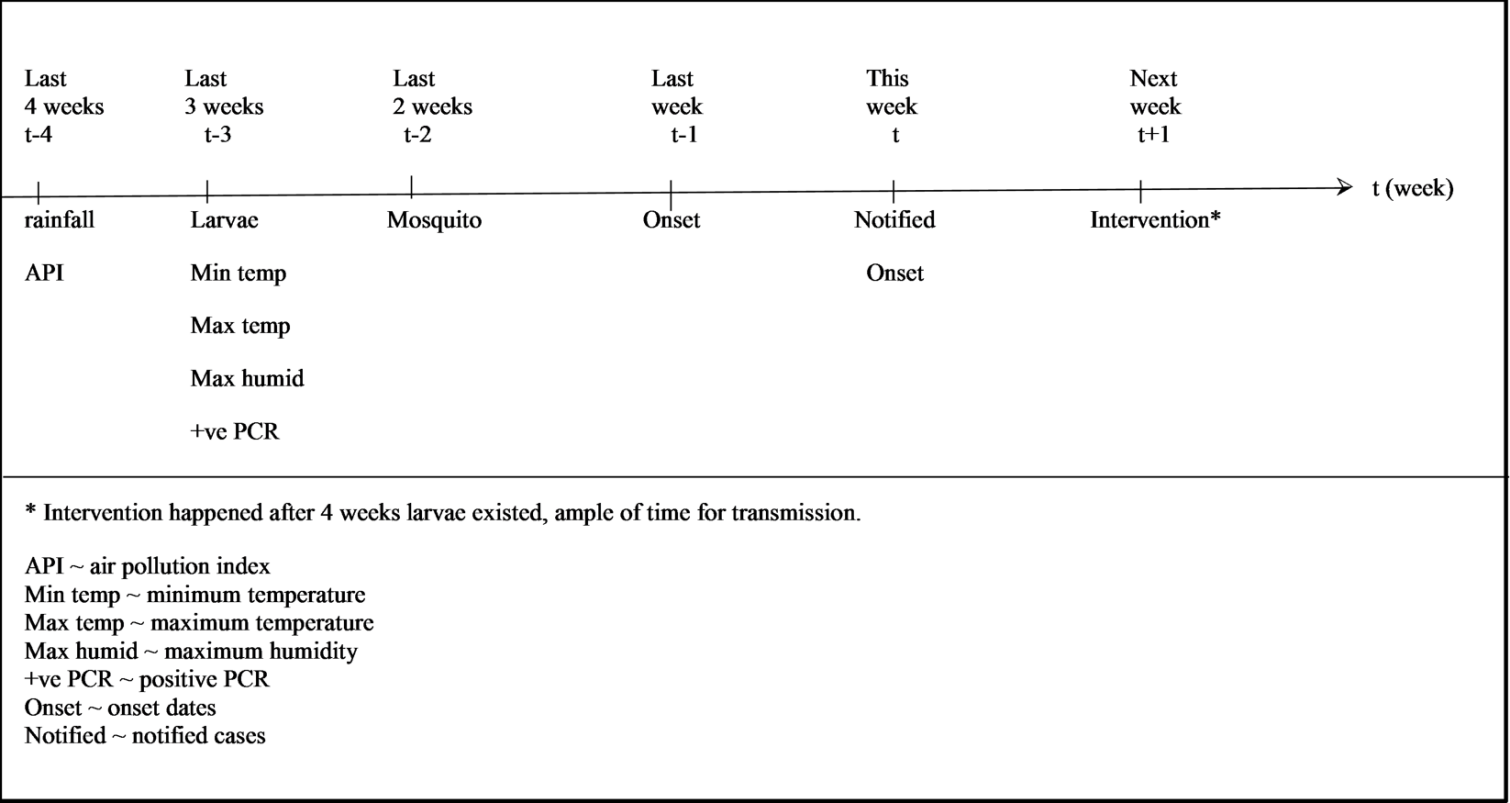
Conceptual relationship: Epidemiological, entomological & environmental factors based on weeks.

Based on the significant correlations, Fig 5 outlined the conceptual relationship among epidemiological, entomological, and environmental factors based on weeks. Notified cases related to this week and last week onset date; last 3 weeks larvae; last 3 weeks positive PCR; last 3 weeks minimum & maximum temperature; last 3 weeks maximum humidity; last 4 weeks rainfall and last 4 weeks air pollution index (API), respectively. Notified cases were also related with next week intervention and conventional intervention only happened 4 weeks after larvae were found, indicating ample time for dengue transmission.

According to Pena-Garcia et al. [17] the density of mosquitoes in an area is not always the best indicator of dengue cases or outbreak. Instead, infection rate in mosquito vector and temperature might explain better such heterogeneity. In addition, weather is a key factor to have in mind in the epidemiological surveillance of dengue, since it affects some mosquito life traits as well as virus replication [31]. Thus, some features such as temperature, precipitation and relative humidity have been associated with mosquito development, survival, density, and oviposition rates [31–33]. On the other hand, virus replication and transmission have been described as temperature-dependent [34–37].

**Fig 6:**
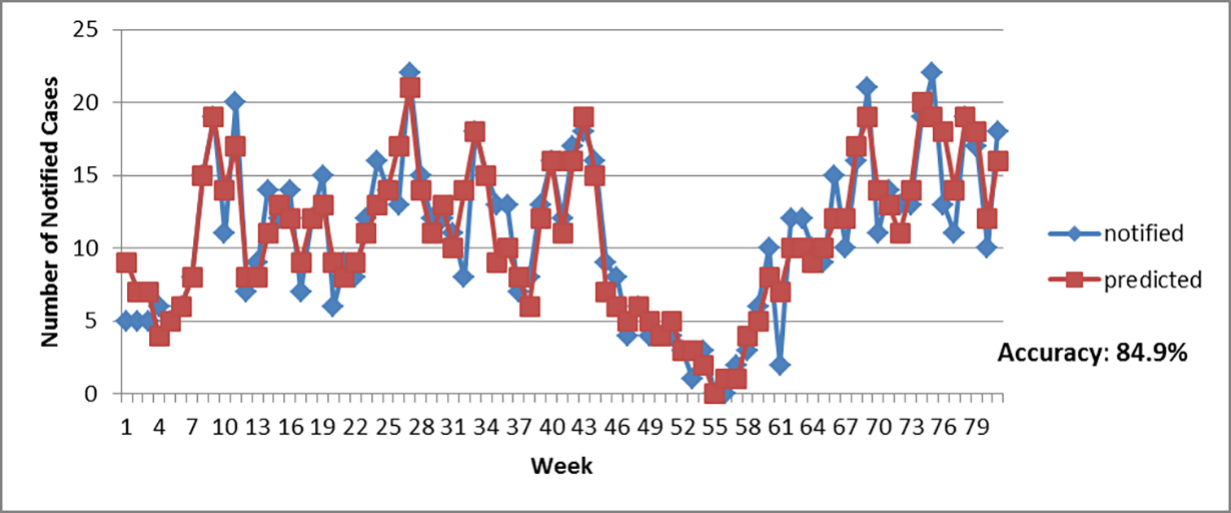
Actual notified cases versus predicted values (Selayang).

**Fig 7:**
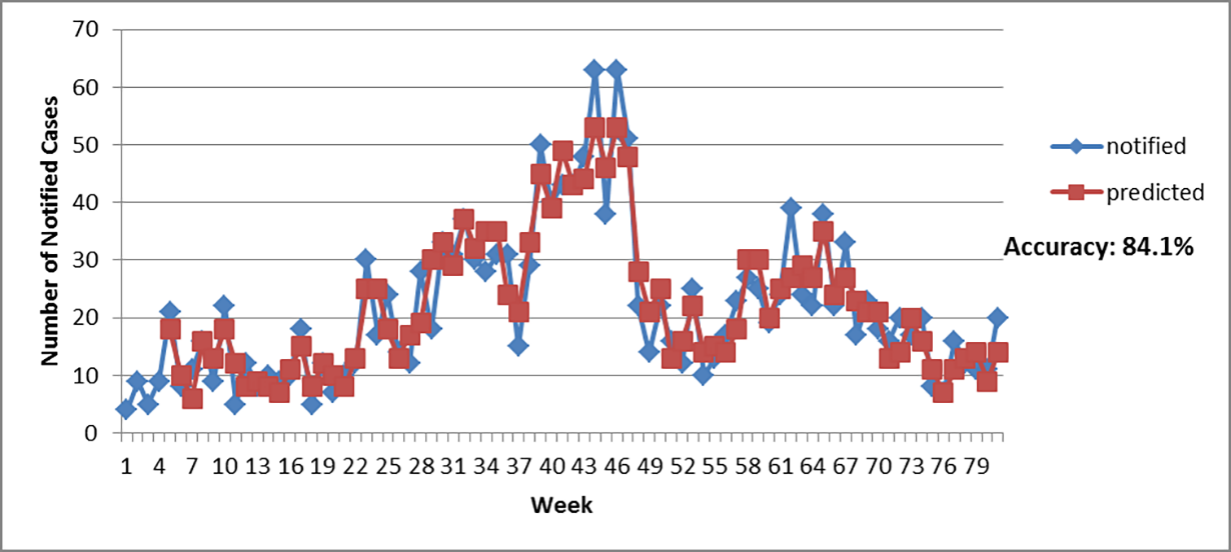
Actual notified cases versus predicted values (Bandar Baru Bangi).

Based on the significant relationship among the three factors (epidemiological, entomological, and environmental). Table 4 displayed the estimated Autoregressive Distributed Lag (ADL) model. Both models (Selayang and Bandar Baru Bangi) have high accuracy which is 84.9% and 84.1% respectively. Figs 6 and 7 visualized the accuracy by plotting the actual notified cases with predicted notified cases based on the ADL model. Hence, these models can be used in predicting dengue outbreaks in the near future and act as an early warning system.

**Table 4:** Estimated Autoregressive Distributed Lag (ADL) model.

Variables (Predictors)	Selayang	Bandar Baru Bangi
Intercept	-18.400	8.138
Onset	0.372***	0.393***
Onset_1	0.381***	0.399***
Larvae_3	0.007***	0.005*
PCR_3	0.624*	0.727
InterventionLD1	0.061	0.058
Rainfall_4	-0.021	0.017
MinTemp_3	-0.272	1.352
MaxTemp_3	0.705	-1.323
MinHumid_3	-	-0.091
MaxHumid_3	0.039	0.051
API_4	-0.016	0.007
Adjusted R^2^	0.8465	0.8388
Information Criterion	135.039	268.466
Accuracy	84.9%	84.1%

***Significant at 1%.

*Significant at 10%.

Target: Notified Cases.

Both of our models reflected similar findings by Ramachandran et a1., [38] and Hii et al., [39], which showed environmental factors (rainfall, temperature and humidity) have a significant role in their dengue forecasting model. However, the advantage of our models is to be able to connect the three factors (epidemiological, entomological and environmental) significantly, thus enhance better understanding of the relationships of the three factors with respect to dengue outbreak in a real world setting.
